# Health Problems with Mycotoxins in Cattle—A Review

**DOI:** 10.3390/molecules31010043

**Published:** 2025-12-22

**Authors:** Lidia Radko, Katarzyna Dudek, Paula Żakowicz, Sebastian Smulski, Roland Kozdrowski

**Affiliations:** 1 Department of Preclinical Sciences and Infectious Diseases, Faculty of Veterinary Medicine and Animal Sciences, Poznan University of Life Sciences, 60-637 Poznan, Poland; 2 Department of Bacteriology and Bacterial Animal Diseases, National Veterinary Research Institute, 24-100 Pulawy, Poland; katarzyna.dudek@piwet.pulawy.pl; 3 Students Scientific Society of Veterinary Medicine, Section of Veterinary Pharmacology and Toxicology “Paracelsus”, Faculty of Veterinary Medicine and Animal Sciences, Poznan University of Life Sciences, 60-637 Poznan, Poland; paulazakowicz@gmail.com; 4 Department of Internal Diseases and Diagnostics, Faculty of Veterinary Medicine and Animal Sciences, Poznan University of Life Sciences, 60-637 Poznan, Poland; sebastian.smulski@up.poznan.pl (S.S.); roland.kozdrowski@up.poznan.pl (R.K.)

**Keywords:** cattle, mycotoxins, emerging mycotoxins, health, mycotoxicosis, adverse effects

## Abstract

Mycotoxins are toxic compounds produced by certain types of fungi that can contaminate animal feed. Cattle may be exposed to these toxins through contaminated feed sources such as cereal grains (e.g., corn, barley), silage, hay, and other roughages, where aflatoxins, fumonisins, T-2 toxins, zearalenone, deoxynivalenol, ochratoxins, and emerging mycotoxins are most commonly found. Cattle are generally less sensitive to mycotoxins, mainly due to detoxification processes occurring in the rumen. The rumen plays a key role in the degradation or transformation of mycotoxins through the activity of ruminal microorganisms and enzymes before these toxins are absorbed into the bloodstream. However, despite this natural defense, mycotoxins have been shown to impact ruminant health. This article aimed to analyze the literature on the negative effects of mycotoxin exposure on cattle health. In January 2025, a systematic search of various databases (PubMed, Google Scholar, EMBASE, and Web of Science) was conducted in Google Chrome to identify studies assessing the association between mycotoxin exposure and health complications in cattle. Symptoms of mycotoxin poisoning are nonspecific and include metabolic and hormonal imbalances, inflammatory conditions, weakened immune response, digestive disorders, reduced productivity, and reproductive issues. These toxins may also compromise the safety of the food chain, including the quality of milk and meat products. Due to the increasing risk of mycotoxin contamination in feed, a comprehensive approach to feed management is essential. This includes regular monitoring, proper storage of raw materials, and the use of plant protection products that minimize the risk of contamination.

## 1. Introduction

Mycotoxins are secondary metabolites of filamentous fungi commonly occurring in feed and plant-derived raw materials, posing a significant threat to livestock health and food safety [1]. In cattle nutrition, mycotoxin contamination of feed represents a global problem, and their presence may lead to adverse health effects, including immunosuppression, reduced productivity, and substantial economic losses in animal production [2,3,4]. Despite intensive research efforts, comprehensive characterization of the mycotoxin profiles present in cattle diets remains challenging, mainly due to the diversity of toxic compounds, their frequent co-occurrence, and limited knowledge regarding their biological interactions [5]. Contaminated feed may simultaneously contain multiple mycotoxins, such as aflatoxins, ochratoxins, fumonisins, deoxynivalenol, zearalenone, and trichothecenes [6,7]. Among the mycotoxins regulated by European Union legislation, exceedances of the maximum permitted levels for aflatoxin B1, zearalenone, and the sum of T-2 and HT-2 toxins have been most frequently reported in recent years. Monitoring data indicate that concentrations of these compounds in feed may reach levels far exceeding regulatory limits, highlighting the scale of the problem and the need for further investigation into their toxicity in ruminants. It should be emphasized that health risks are not limited to exposure to individual mycotoxins. Numerous studies have confirmed a high frequency of co-occurrence of mycotoxins in feed, and the simultaneous presence of multiple compounds may result in additive or synergistic effects, even when the concentrations of individual mycotoxins do not exceed established regulatory thresholds. Co-occurrence is particularly common for mycotoxins produced by fungi of the genus *Fusarium*, such as fumonisins, deoxynivalenol, and zearalenone. Despite the growing number of reports on mycotoxin co-occurrence, data on their mutual interactions and their impact on cattle health remain limited. An additional challenge in risk assessment is the occurrence of mycotoxins in conjugated forms, referred to as masked or bound mycotoxins, which may exhibit different bioavailability and toxicity compared to their parent compounds [8]. Although ruminants are generally considered less sensitive to mycotoxins than monogastric animals—primarily due to the ability of rumen microflora to biotransform certain toxins into less harmful metabolites—these mechanisms do not provide complete protection [9,10,11,12,13,14]. Prolonged exposure to contaminated feed may impair rumen function, induce immunosuppression, negatively affect reproductive performance, and reduce milk and meat production. Numerous clinical cases and in vivo studies reported in the literature document the detrimental effects of mycotoxins on cattle health, including gastrointestinal disorders, nephrotoxicity, hepatotoxicity, lameness, and increased susceptibility to infectious diseases [15,16,17,18]. Particularly severe outcomes have been observed in cases of simultaneous exposure to multiple mycotoxins, which may lead to chronic, debilitating clinical symptoms and even animal mortality [19]. Moreover, certain mycotoxins and their metabolites may be excreted in milk [20,21,22,23] or accumulate in tissues [24], posing a direct risk to consumer health, especially for children and immunocompromised individuals. Considering the resistance of mycotoxins to technological processing and the lack of established maximum limits for many of these compounds in products of animal origin, this issue is of major importance for both public health and the economics of milk and beef production [25,26,27,28,29]. Effective risk assessment requires consideration of the combined effects of multiple mycotoxins as well as their impact on ruminant metabolism and immune function.

This review aims to provide a concise and comprehensive overview of the current state of knowledge regarding the effects of the most commonly occurring mycotoxins in feed on cattle health, with particular emphasis on in vivo studies and clinical observations. This work highlights the role of mycotoxins as contributing factors in the development of nutritional imbalances, metabolic disorders, and productivity losses, and identifies key research gaps that warrant further investigation.

## 2. Literature Review Method

### 2.1. Search Strategy and Data Sources

The electronic databases (PubMed, Google Scholar, EMBASE, and Web of Science) were searched for peer-reviewed articles published between 1974 and 2025 using the following keywords: “mycotoxins and cattle health”, “aflatoxins and cattle health”, “zearalenone and cattle health”, “trichothecenes and cattle health”, “fumonisin and cattle health”, “emerging mycotoxins and cattle health”, “mycotoxins and detoxification”, “mycotoxins and cattle feed”. Studies identified during the electronic database search were combined, duplicates were removed, and articles were analyzed for their relevance to the review based on the information contained in the title and abstract. Abstracts were analyzed and potentially eligible articles were identified.

### 2.2. Inclusion/Exclusion Criteria

Studies were included if (A) publications provided toxicological, nutritional, and phytochemical data regarding mycotoxins and their effects on cattle, (B) publications sufficient experimental or observational data, (C) they contained quantitative results, (D) they were published in English. Studies were excluded if they were not directly related to mycotoxins and their impact on cattle health.

### 2.3. Study Selection and Data Extraction

**Figure 1:**
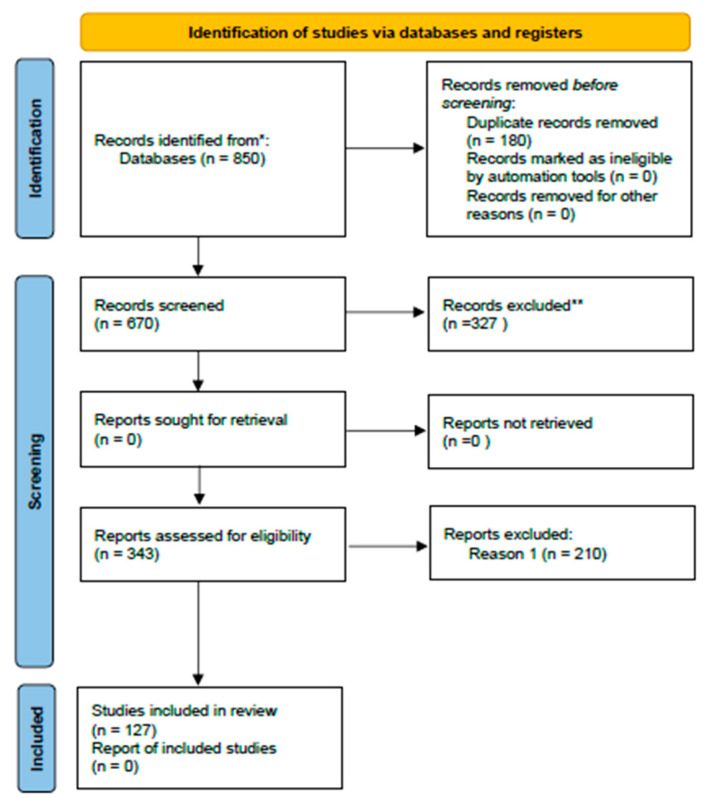
Flowchart of methodology. * The reported the number of records identified from all database or register searched. ** The indicated how many records were excluded by a human.

The Systematic Review follows the general approach of PRISMA 2020 statement. The full text of references identified as potentially relevant was obtained, and papers were included by applying the inclusion criteria. The number of documents retrieved was 850. Of those, 180 were duplicates and were removed, and 343 papers were excluded based on the criteria used. The number of studies finally included in this systematic review was 127 (Figure 1).

## 3. Impact of Mycotoxins on Cattle

### 3.1. The Effect of Aflatoxins on Cattle

**Figure 2:**
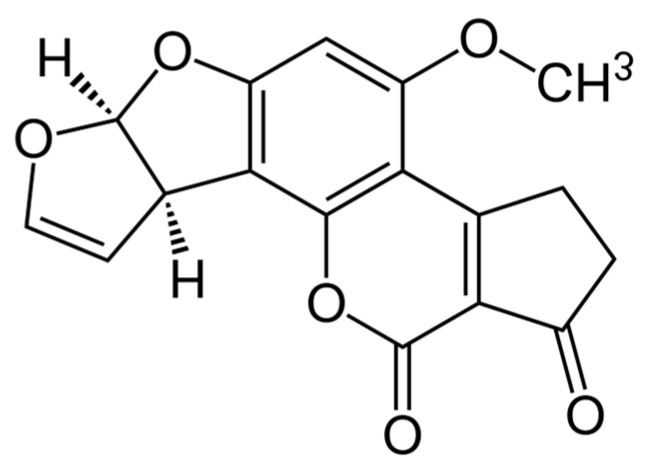
Chemical structure of aflatoxin B1.

Aflatoxins are a group of structurally related difuranocoumarin compounds (Figure 2) produced primarily by *Aspergillus flavus*, *Aspergillus parasiticus*, and *Aspergillus nomius* [30]. These toxins are found worldwide, but they are produced more frequently in warm, tropical, or subtropical climates.

Aflatoxins may be present on starchy cereal crops, peanuts, or cottonseed before harvest and can also be found during post-harvest storage. Due to their hepatotoxic, immunosuppressive, mutagenic, and carcinogenic properties, these mycotoxins pose a significant public health risk. In cattle, clinical effects of aflatoxins include hepatopathy, increased concentrations of bilirubin and hepatic enzymes in the blood, prolonged coagulation time, and reduced immunity, which may manifest as vaccine ineffectiveness or inadequate responses to antibiotic therapy. The impact of these mycotoxins can also negatively affect weight gain and feed conversion ratios. Aflatoxins influence cellular immunity, cytokine production, and non-specific humoral factors, including interferon, complement, and certain bactericidal components of blood serum [31]. The permissible concentrations of aflatoxins in animal feed are defined by legal regulations (Table 1) [25].

*A. flavus* strains primarily produce aflatoxin B1. This species of fungus usually invade aging or damaged plant tissues. The B1 aflatoxin is commonly present in the highest concentration and is considered the most toxic and oncogenic among all aflatoxins. *A. parasiticus* strains may produce B1, B2, G1 and G2 aflatoxins, which fluoresce blue or green are observed under UV light. M1 and M2 are 4-hydroxylated metabolites of B1 and B2, respectively. They may occur in the tissues, milk and dairy products from animals that have consumed feed contaminated with aflatoxins. Aflatoxins have been classified as a group 1 carcinogen by the International Agency for Research on Cancer (IARC) [31]. Its presence is associated with the development of hepatocellular carcinoma in humans. Sterigmatocystin, a mycotoxin produced by molds, particularly by *A. versicolor*, serves as a precursor to aflatoxins synthesis. This type of toxin is a hepatotoxin and carcinogenic agent and may induce clinical poisoning similar to aflatoxins, but with lower efficacy.

Aflatoxins are lipophilic compounds with low molecular weight that are passively absorbed from gastrointestinal tract. It is important to note that the absorption of aflatoxin may occur in the oral cavity or esophagus of the dairy cows, even before they enter the rumen [32,33]. Toxins from the gastrointestinal tract enter the hepatic portal system. The absorption of aflatoxins takes less time in young animals than in older ones. The results of studies on microbiological degradation of aflatoxins in the rumen are inconclusive. Aflatoxins may be minimally destroyed in the rumen, but the degree of aflatoxin degradation is influenced by the type of feed ration, rumen incubation time, feeding time, animal species and individual predispositions [33,34,35]. B1 aflatoxin may reversibly bind to albumins circulating in blood, while unbound B1 aflatoxin is transported from the vessels into the tissues. Aflatoxins do not accumulate in tissues, but multiple exposure may generate toxic consequences in organs. Aflatoxins may pass through the placenta and damage fetal tissues. However, there are not many studies focusing on reproductive effects. Elimination of aflatoxins from the body is possible through bile, urine, feces and milk. The majority of animal species eliminate aflatoxins within 24 h after exposure [36]. As a result of biotransformation, which takes place mainly in the liver, several metabolites are formed with the activation of cytochrome P450: CYP1A2 and CYP3A4, resulting in the formation of 8,9-epoxide. Biological transformation of toxins can also occur in the kidneys, gastrointestinal tract, and other organs, but to a much lesser extent. Covalent binding of aflatoxin 8,9-epoxide to DNA, RNA, and proteins indicates the formation of tissue adducts, which disrupt the synthesis of DNA, RNA, and proteins and harm cellular processes, which may lead to disturbances in organ functions. Potential results include immunosuppression (both humoral and cellular immunity), cell death, mutagenesis and neoplasia. Other metabolites (e.g., AFM1, AFM2, AFQ1, aflatoxicol) exhibit lower toxicity. The conjugation of these products with sulfate and glucuronide is a detoxification reaction. The aflatoxicol is an oncogenic factor and may be oxidized to B1 aflatoxin. The aflatoxicol has been identified in milk, fermented dairy products, and tissues. The pasteurization and ultra-pasteurization of milk with varying fat content do not destroy this type of toxin [36]. M1 aflatoxin is the major product excreted in milk and urine. It is possible to monitor the toxin in relation to exposure. M1 appears in milk as early as 5 min after ingestion of B1 [37]. The excretion of M1 in milk is influenced by the species of the animal, lactation status, and feeding practices, particularly the time elapsed from exposure to the toxin-contaminated feed to milking. For example, when concentrates were primarily fed at night, the concentration of M1 was higher in morning milk in comparison to evening milk. A seasonal dependency related to feeding practices was also observed—M1 aflatoxin levels in milk were higher during the winter when cows were fed larger amounts of concentrates than in the summer period. The concentration of M1 in milk approaches baseline levels approximately 3 to 6 days after the daily consumption of aflatoxins, with M1 becoming undetectable in milk within 2 to 4 days. While all animals are susceptible to aflatoxins, sensitivity varies by species. Young individuals and monogastric animals are particularly vulnerable to toxicity. In dairy cattle, toxicosis primarily manifests as reduced milk production and exceeds the allowable residue levels of this substance in milk. The total dietary aflatoxin concentration for dairy cattle and immature ruminants should not exceed 20 ppb (Table 1). In the European Union, the maximum permissible level for AFM1 in milk is typically set at 0.05 µg/kg. In the United States, the FDA has established action levels for AFM1 at 0.5 µg/L (or 0.5 µg/kg in milk). However, legal regulations in the EU provide maximum levels of aflatoxins and other mycotoxins in feed (Table 2) [38].

Cattles fed aflatoxin-contaminated feed may exhibit reduced feed intake and lower body weight gains. Clinical signs of chronic aflatoxicosis in cattle include reduced appetite and feed conversion, lower milk production, and noticeable signs of jaundice. Hepatic enzymes are usually elevated and prothrombin time may be prolonged in the blood results. A sensitive indicator of aflatoxicosis is decreased productivity, which is associated with nutritional interactions, anorexia, altered protein and lipid metabolism in the liver and disturbances in hormonal metabolism [39,40,41]. Levels of aflatoxins are particularly high during heatwaves and in damaged field crops. It is particularly concerning that they can be concentrated in grain by-products, such as dried distiller grains (DDGs), potentially increasing their toxicity by up to three times. Acute lethal aflatoxin poisoning in a cow has been reported following the feeding of moldy sweet corn containing more than 2300 ng of aflatoxin/g (ppb) of corn [42]. Sweet corn is considered a source of aflatoxins due to its higher sugar content, which is necessary for mold growth. Veterinarians should consider multiple sources of aflatoxins in feed rations and the conditions in which grains are stored on the farm when analyzing mycotoxin poisoning [43].

Diagnosis of aflatoxicosis in cattle is based on the association of clinical signs with the detection of high levels of mycotoxins in the feed. Multiple analytical methods may be used to determine aflatoxins in feed. It should be emphasized that positive grain fluorescence under black light (blue-green color at 365 nm) suggests the presence of *Aspergillus* metabolites but not aflatoxins.

Treatment of aflatoxicosis in cattle is based on ensuring an optimal amount of vitamins and micro- and macro-elements (especially protein) in the diet. To prevent aflatoxin production after harvest, it is recommended to store cereal grains at humidity below 12% and oilseeds below 8–9% over a wide temperature range. Cereals and oil seeds should be stored in clean, weatherproof places. Cleaning and removal of damaged grains can reduce the level of mycotoxins. There are several methods to reduce aflatoxin contamination of feeds, including the use of mold inhibitors, physical separation, fermentation, roasting, irradiation, ozonation, ammonium treatment and the use of mycotoxin adsorbents. None of the mentioned methods is without disadvantages, such as non-hundred percent effectiveness, high cost and impossibility of large-scale application. It was reported that treating grain with anhydrous ammonia may reduce aflatoxin level by approximately 30%. There are commercially available products that function by sequestration or binding of mycotoxins, as well as reducing their absorption from the animal’s gastrointestinal tract. These preparations include calcium and sodium silicates, bentonite, activated carbon, esterified glucomannan, various clays and yeast cultures. A few of these compounds have the potential in partially binding B1 aflatoxin, thereby decreasing the contamination of milk with M1 aflatoxin [44,45,46,47,48]. Positive responses regarding the reduction in M1 in milk are not consistent across all binding substances. Reference publications indicate that some clay products only partially bind aflatoxins in ruminants due to competition with unknown ruminal compounds for binding sites on the clay. Including calcium and sodium aluminosilicates or clay products at low concentrations in the diet seems to be a reasonable investment in the partial sequestration of aflatoxins, and as a result, reducing potential toxicity and residues of substances in milk in lactating female cattle. The use of yeast products may stimulate immune responses and partially compensate for some of the adverse effects, but it does not appear to significantly decrease toxicity or milk residues.

### 3.2. The Effect of Ochratoxins on Cattle

**Figure 3:**
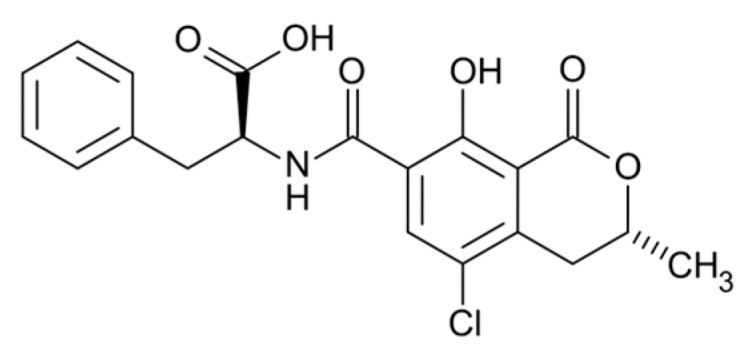
Chemical structure of ochratoxin A.

Ochratoxins (OT) are phenylalanine-dihydroisocoumarin compounds (Figure 3) produced primarily by *Aspergillus* spp. and some *Penicillium* spp., including *A. ochraceus*, *A. carbonarius*, *A. alliaceus*, *A. auricomus*, *A. niger*, *A. melleus*, *A. glaucus*, *P. verrucosum and P. nordicum* [49]. OT toxins are usually produced when stored, contamination of grains before harvest is also possible [50]. Ochratoxins are present in the seeds of many plants and food products. They have been detected in rye, wheat, barley, corn, sorghum, millet, rice and oilseeds (Table 2) [49,51]. The most common forms of OT are ochratoxins A (OTA), B (OTB) and C (OTC), which differ in chemical structure and toxicity. OTB is the non-chlorinated form of OTA, while OTC is the ethyl ester of OTA formed in the presence of ruminal fluid [49,50]. OTA is the most prevalent and toxic form of ochratoxin, whereas OTB exhibits the lowest degree of toxicity [49]. Ochratoxins exhibit immunosuppressive and nephrotoxic effects. Due to their potentially carcinogenic properties, the IARC has classified OTA as a group 2B agent, that is, a possible carcinogenic agent [31]. In addition, ochratoxins inhibit protein synthesis and affect the activity of humoral immune factors, particularly immunoglobulins, as well as lead to a reduction in NK cell activity. Ochratoxins may also induce oxidative stress [50]. Regarding the chemical structure of ochratoxins, each of them contains a nonpolar end and several polar groups in the side chain (Figure 3). They can bind to lipids and proteins, such as plasma albumin [52]. Analogously to aflatoxins, ruminants exhibit definitely lower sensitivity to OTA compared to monogastric animals. Rumen microorganisms, primarily protozoa, convert OTA into non-toxic OT. This process affects the half-life of OTA, which ranges from 0.6 to 3.8 h. After 10 to 24 h, the mycotoxin is completely metabolized [53]. It is significant to note that high-protein feed reduces the detoxification rate of OTA in both calves and adult individuals. Most OTA is eliminated in the urine. OTA residues have not been detected in meat and other animal tissues from cattle. However, OTA is found in the tissues of pigs (mainly the kidneys) and poultry. OTA residues detected in milk are at low levels [54,55,56] and only in situations of chronic exposure of cattle to this type of toxin. After a single exposure to a high dose of ochratoxin A (13 g/kg body weight), diarrhea, coordination disturbances, anorexia, and decreased milk production were observed. All of these symptoms were temporary [55,57]. It should be mentioned that the toxic effect of ochratoxin occurs in the case of chronic exposure to low doses, as evidenced by clinical cases reports of poisoning in cattle.

Feeding cattle with feed contaminated with OTA (3 mg/kg body weight, up to 6 ppm) resulted in clinical symptoms of uremia. In addition, signs such as severe diarrhea, dehydration, anorexia, depression, and hypothermia were observed. In many cases, the intoxication led to lethal consequences [56]. Anatomopathological and histological changes in the kidneys indicated strong nephrotoxic effects of OTA. Renal fibrosis, presence of hyaline casts, proximal tubule damage, and tubular dilatation were noted. Furthermore, fatty changes were observed in the liver.

Human exposure to OTA results mainly from the consumption of contaminated cereals; however, an indirect source of OTA exposure may be the consumption of products of animal origin derived from animals fed contaminated diets. The low incidence of OTA and its generally low concentrations in edible tissues may suggest that food of animal origin is unlikely to pose a significant risk to adult consumers.

### 3.3. The Effect of Trichothecenes on Cattle

**Figure 4:**
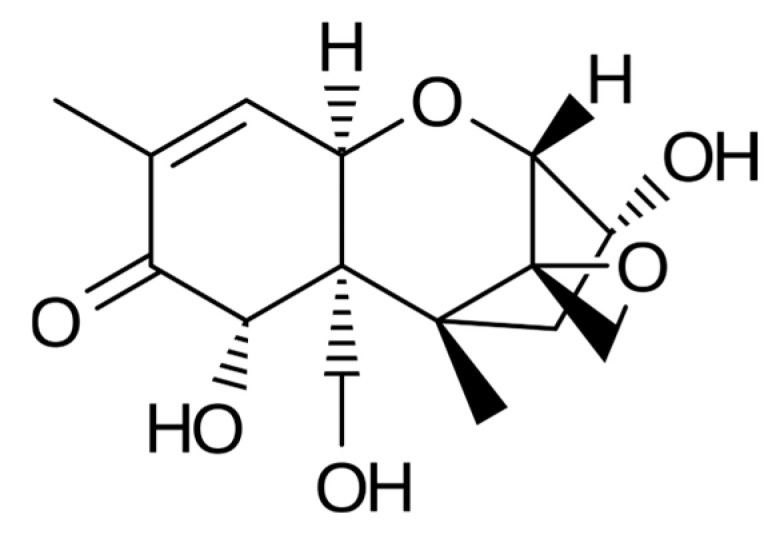
Chemical structure of DON.

**Figure 5:**
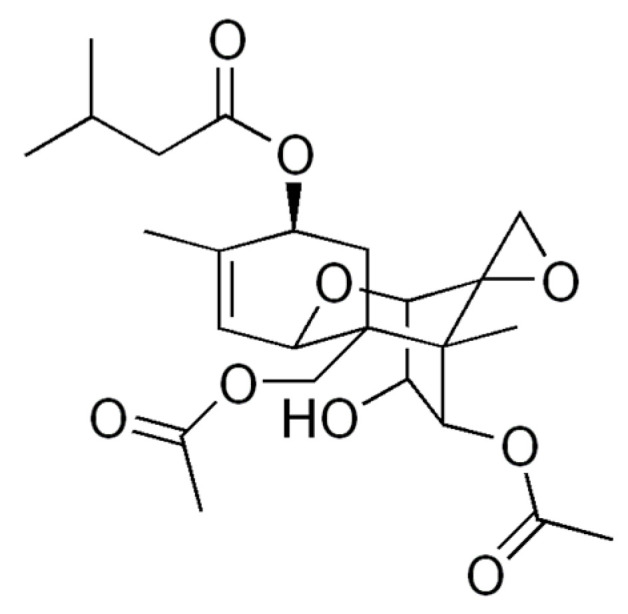
Chemical structure of T-2 toxin.

Trichothecenes are several groups of mycotoxins produced by fungi of the genus *Fusarium* [58]. They belong to sesquiterpenoid compounds (Figure 4 and Figure 5) with an epoxide group at the C12–13 position, which is considered essential for their toxicity. Four groups are distinguished among trichothecenes: type A, B, C and D. Type A group includes: T-2 toxin, HT-2 toxin, diacetoxyscirpenol (DAS), monoacetoxyscirpenol (MAS) and neosolaniol (NEO). Type B group includes: deoxynivalenol (DON), nivalenol (NIV), fusarenon X and DON derivatives: 3-acetyldeoxynivalenol (3-Ac-DON) and 15-acetyldeoxynivalenol (15-Ac-DON) [59]. C-type trichothecenes contain a C-7/C-8 epoxide (e.g., crotocin), whereas D-type trichothecenes have an additional ring connecting the C-4 and C-15 positions (e.g., roridin A, verrucarine A, and satratoxin H) [58,59]. A-type trichothecenes and B-type trichothecenes are most commonly found in barley, wheat, oat, and corn (Table 2). Studies have shown that the toxicity of A-type trichothecenes is greater than that of the B-type group. In warmer climates, it has been observed that type A trichothecenes occur in lower concentrations than type B trichothecenes in contaminated cereals [58,59,60]. Veterinary medicine focuses on a relatively small number of trichothecenes, although more than 100 have been identified. Trichothecenes are very stable mycotoxins that remain in feed for many years at high levels. Health problems on cattle farms have been most often associated with DON feed contamination. Most clinical cases are chronic or subchronic. Long-term exposure to low levels of DON in the cattle diet is associated with nutritional disorders, reduced milk/meat production, and a weakened immune system. However, it should be noted that ruminants are less sensitive to DON compared to monogastric animals and young animals (calves).

Comparing the toxicity of the trichothecenes, it was shown that the toxicity of DON, 15-AcDON, fusarenon-X, and nivalenol was at a similar level, whereas 3-AcDON exhibited lower toxicity [60,61]. Animal studies have demonstrated that 15-AcDON exhibits higher cytotoxic activity compared to 3-DON and DON, particularly in relation to cell proliferation, intestinal barrier integrity, and intestinal structure. The mechanism of action of this toxin on enterocytes is associated with an enhanced capacity for activating mitogen-activated protein kinase [62,63]. There are several mechanisms of the toxic effects of trichothecenes on cells. They include: inhibition of protein, RNA, and DNA synthesis, alteration of membrane structure and mitochondrial function, stimulation of lipid peroxidation, hypoxia and oxidative stress, induction of cell death, and activation of cytokines and chemokines [62,63,64]. The earliest clinical sign of trichothecene poisoning that can be observed in a herd is a significant decrease in the animals’ feed consumption. If there is no intervention related to changing the feed, poisoning may occur. After dietary exposure to trichothecenes, diarrhea is frequently observed in animals. It results from impaired intestinal absorption and impaired permeability of nutrients caused by morphological and functional damage to the mucous membrane of the intestines [65,66]. Korostoleva et al. showed that feeding dairy cows with feed containing DON led to an increase in Na^+^ levels in serum, a decrease in leukocyte phagocytic activity, and changes in immunoglobulin concentrations [67]. Feeding animals a diet contaminated with DON (8–12 mg/kg) for 90 days resulted in reduced dry matter intake, impaired rumen protein metabolism, decreased milk yield, and changes in milk quality [68]. Experimental studies in dairy cows have shown that feeding feed contaminated with DON leads to appetite disturbances, ulceration of the rumen and reticulum mucosa, reduced milk production, increased somatic cell count in milk, and worsened reproductive performance. DON also causes disturbances in rumen fermentation and reduces the amount of digestible protein reaching the duodenum.

The metabolism of trichothecenes takes place in the digestive system of ruminants before the compounds are absorbed into the blood. The metabolism of DON in the rumen leads to the formation of deepoxy DON. Deepoxidation of DON is one of the stages of trichocene deactivation, which results in the formation of a less toxic compound. The next phase of trichothecene metabolism in the rumen is deacetylation, which occurs with the participation of ruminal microorganisms, mainly protozoa. The absorbed part of DON is also metabolized in hepatic microsomes [69]. Crucial for preventing poisoning of cattle by DON and its acetylated derivatives is maintaining a ruminal microbiota homeostasis. For this purpose, animals should be provided with an appropriate amount of bulk feed. A clinical case in Belgium demonstrated that feeding a feed concentrate containing considerable levels of deoxynivalenol (DON, 1.13 mg/kg feed) induced severe liver failure in 2- to 3-month-old beef calves. Consequently, the toxicity of DON in calves is closely related to roughage provision and the associated stage of ruminal development [70]. A diet for cattle that does not include bulk feed should contain lower levels of DON in the feed ration (2 ppm). Low levels of DON (below 5 ppm) in the feed do not affect feed intake, weight gain, reproduction, lactation period or beef quality [71,72]. Moreover, it was observed that beef cattle are more tolerant of higher levels of DON in the feed in comparison to dairy cattle [63]. That difference in sensitivity is explained by the higher stress in dairy cows and increased dry matter intake, faster rumen turnover and reduced rumen microbial degradation time in dairy cows [64]. Feeding dairy cows with DON (12 ppm) during lactation did not reveal any residues of trichothecene (<1 ng/mL or ppb) DON or de-epoxy DON in milk [73]. No effect of DON on feed intake and milk production by dairy cows was noted. Twenty-four hours after administering high doses of DON to lactating cows, the toxin was detected in their serum and milk. It is estimated that 0.0001% of the administered dose of DON transfers to the milk [74]. Low concentrations of DON in edible tissues and milk suggest that food of animal origin does not pose a health risk to consumers.

T-2 and HT-2 toxins are some of the most toxic trichothecenes. Clinical symptoms of poisoning with these types of toxins in cattle include low appetite, weight loss, diarrhea, disruptions in immune system function, coagulopathy and hemorrhage and cellular necrosis of mitotically active tissues such as the skin, intestinal mucosa, spleen, bone marrow, ovaries and testes [75]. Ruminants can rapidly deacetylate T-2 toxin to HT-2 toxin. It is complicated to distinguish the clinical effects of T-2 toxin from HT-2; therefore, in the analysis of poisonings, the concentrations of both toxins are added. Decreased feed consumption was observed in calves of beef breeds, which were given T-2 toxin in the feed at a dose of 0.3 mg/kg for 6 weeks [76]. In calves given a twice higher dose, 0.6 mg/kg, anorexia, weight loss and diarrhea were observed. Clinical effects of field poisoning in dairy cows included anorexia, elevated temperature and abortions. In addition, an approximately 20% increase in neonatal deaths was observed, which was associated with feeding mothers with moldy corn [77]. Analysis of the corn revealed the presence of the fungi *F. tricinctum*, *F. roseum* and *F. moniliforme* as well as *Penicillium* spp. Although low concentrations of T-2 toxin were detected, it was hypothesized that the poisoning was caused by toxic interactions with other mycotoxins present in the feed.

The levels of type A trichothecenes (sum T-2 +HT-2 toxins, 5 ppm) in feed do not result in symptoms of poisoning in calves; however, they should be eliminated in feed for pregnant and lactating females. It has been shown that T-2 toxins can cross the placenta and lead to abortions. The risk of a threat to public health from residues in edible tissues and milk with T-2 toxin has not been assessed so far, probably with rapid metabolism and excretion from the body [33].

In trichothecene poisoning of cattle, the sublethal effects of low doses of trichothecenes should be considered. In the evaluation of poisoning cases, it is essential to assess not only the feed but also the animals’ feeding practices, herd management practices, past illnesses and environmental conditions. Rejection of contaminated feed from the diet improves prognosis. The conditions of plant storage, grain storage (humidity below 13%) and hay/straw storage (humidity below 20%) play a significant role in preventing poisoning in order to limit the production of trichothecenes. However, the use of adsorbents (bentonites, aluminosilicates and some zeolites) to bind trichothecenes in feed is problematic. It has been shown that some b-glucans and mannans have toxic potential [78]. The combination of several adsorbents (mineral and organic) seems to be more effective in counteracting the adverse effects of several mycotoxins in feed. The use of sodium metabisulfite with propionic acid reduced deoxynivalenol contamination of moist cereal grains [79].

### 3.4. The Effect of Zearalenone on Cattle

**Figure 6:**
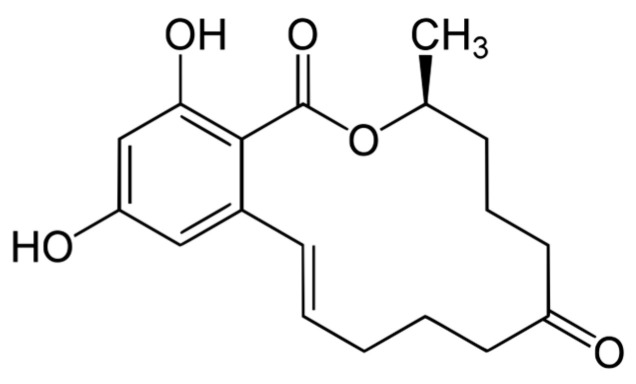
Chemical structure of zearalenon.

*Fusarium* fungi are not only a source of trichothecenes but also of the estrogenic mycotoxin zearalenone (ZEA) and is a macrolide comprising a fourteen-membered lactone fused to 1,3-dihydroxybenzene (Figure 6). This type of toxin commonly occurs in cereals, i.e., corn, wheat, barley, oats or rye (Table 2). ZEA is most often found together with DON [80]. In grain contaminated in the field, low concentrations of zearalenone are found, which increase as a result of improper storage conditions (humidity above 30%) [80,81].

Zearalenone is a nonsteroidal mycotoxin with estrogenic activity produced by several species of Fusarium fungi, primarily *F. graminearum*, but also by *F. culmorum*, *verticillioides (moniliforme)*, *sporotrichioides*, *semitectum*, *Equiseti*, and *oxysporum*. Zearalenone may occur in the parent form or in reduced metabolites a- and b-zearalenol, a- and b-zearalanol, zearalanone and the conjugated metabolites zeralenone-14-O-b-glucoside, zearalenone-16-O-b-glucoside, and zeralenone-14-sulfate [48].

Zearalenone is well absorbed from the gastrointestinal tract. However, its bioavailability is relatively low due to metabolism occurring in the rumen, intestinal cells and liver [81,82]. ZEA penetrates into reproductive cells (ovary cells, testicles) and adipose tissue [83]. The variations in interspecies sensitivity to zearalenone are associated with differences in biotransformation in the liver. The presence of microorganisms in the rumen, which convert it to a- and b-zearalanol, has a significant effect on the metabolism and toxicity of ZEA in ruminants [81,82,84]. B-zearalanol is less toxic, which results in the lower sensitivity of ruminants to this mycotoxin [84]. Although the liver plays a significant role in glucuronidation, the intestinal mucosa is also active in this process. Metabolites of ZEA are excreted in bile and urine, but they may also undergo enterohepatic recirculation and be excreted in feces. Zearalenone and its metabolites may interact directly with the cytoplasmic receptor that binds to 17b-estradiol and causes translocation of the receptor site to the nucleus [82]. Stimulation of RNA in the nucleus leads to protein synthesis and clinical manifestations of estrogenism. Zearalenone has low acute toxicity but may cause hyperestrogenism and fertility disorders. Clinical signs of increased estrogen include prolonged or absent estrus, vulvar edema, increased vaginal discharge, mammary hypertrophy, increased incidence of pseudopregnancy, abortions, infertility and decreased libido. It has been shown that females are more sensitive to the ZEA. The occurrence of abortions in females in clinical trials has been associated with the presence of *Fusarium* fungi in the feed. However, this symptom was not observed after the administration of zearalenone [84,85]. Exposure to ZEN may reduce sperm motility and increase phagocytosis of sperm by immune cells, which may negatively affect fertility in cattle [86]. Long-term feeding of cows with feed contaminated with high levels of ZEA may lead to the presence of this mycotoxin in milk, which could pose a risk to public health [5,87]. Similarly to other mycotoxins, eliminating contaminated feed from the diet leads to an improvement in health condition. In accordance with EU regulations, the maximum content of ZEA in complete feed for calves and dairy cattle has been set at a level of 500 µg/kg (ppb) [88].

### 3.5. The Effect of Fumonisin on Cattle

**Figure 7:**
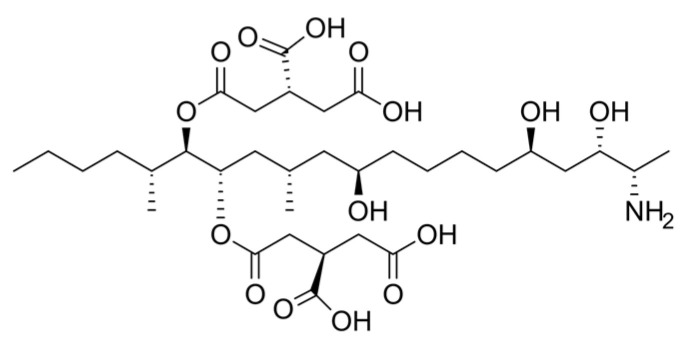
Chemical structure of fumonisin.

Fumonisins are a group of mycotoxins most frequently detected in maize or cereal grains (Table 2). These toxins are produced by fungi of the genus *Fusarium*, including *F. verticillioides (syn. F. moniliforme)*, *F. proliferatum*, *F. fujikuroi*, and other closely related species [89]. It has also been demonstrated that, for example, *Aspergillus niger* produces fumonisins B2 and B4 [90]. To date, six fumonisin forms have been identified: FA1, FA2, FB1, FB2, FB3, and FB4. Among them, FB1 exhibits the highest toxicity [88] and has been classified by the International Agency for Research on Cancer (IARC) as a “possible carcinogen” (Group 2B). Fumonisins are polar compounds and are thermally stable. Chemical analyses describe them as aliphatic hydrocarbons with a terminal amino group and tricarboxylic acid side chains (Figure 7). Their structure resembles that of sphingolipids found in cell membranes [91]. Ruminants appear to exhibit tolerance to fumonisins. In ruminants, ruminal degradation occurs at a level of approximately 8–10%, whereas microsomal metabolism of these compounds is very limited. Fumonisins are primarily excreted in the feces. This group of mycotoxins exhibits cytotoxic and carcinogenic potential [92]. Fumonisins are capable of inhibiting cell growth and differentiation and suppressing the activity of enzymes involved in the conversion of sphinganine to sphingosine. Their main mechanism of action appears to be associated with inhibition of ceramide synthase, which leads to disturbances in sphingoid base metabolism. An altered sphinganine-to-sphingosine ratio is considered a reliable biomarker of exposure to fumonisins. Calves fed diets containing these mycotoxins showed increased liver enzyme activity and mild hepatic changes, without affecting growth rate, performance, or overall health status [93]. The presence of combined concentrations of fumonisins B1 and B2 in feed resulted in reduced milk yield and quality, decreased feed digestibility, and impaired immunity in dairy cows [94]. Analysis of FDA guidelines indicates that lower fumonisin concentrations are recommended in feed for dairy cows than for beef cattle. This is related to the higher sensitivity of dairy cows to mycotoxins, manifested by reduced milk production and decreased appetite [94,95,96]. Only a few, relatively old studies have evaluated the presence of FB1 in milk, some of which demonstrated that fumonisins can be transferred to bovine milk at low concentrations. Because FB1 and FB2 in milk samples are resistant to heat treatment such as pasteurization (62 °C/30 min) and to storage at 4 °C for up to 11 days, the presence of these contaminants constitutes a significant public health concern.

### 3.6. The Effect of Emerging Mycotoxins on Cattle

In the past few years, there has been increased scientific and clinical interest in emerging mycotoxins, which include: enniatins (ENN), beauvericin (BEA), fusaproliferin (FP), moniliformin (MON), roquefortin C, STER, aurofusarin (AUR), bikaverin, culmorin, 15-hydroxyculomorin, tenuazonic acid (Alternaria metabolite), sterigmatocystin (Aspergillus metabolite), and mycophenolic acid (MPA) [1,97]. At present several ENN analogs have been identified: A, A1, B, B1, B2, B3, B4, D, E, F and G [98]. The most common emerging mycotoxins found in feed are: ENN A, A1, B and B1 [98]. Enniatins and beauvericin are produced primarily by fungi of the *Fusarium* genus. Emerging mycotoxins, although found in feed, are not subject to control or legal regulation. They are most often found in cereal grains, including wheat, barley, oats and corn, as well as in silage [98,99]. The most frequently detected emerging mycotoxins in corn silage were emodin (EMO), culmorin, enniatin B1, enniatin B and beauvericin [99]. Among the detected ENNs, enniatin B is most frequently detected in cereal grains, mainly from European countries [100], including Spain [101], Denmark [102], and Finland [103].

The conducted studies on ENN B have shown its ionophore properties, its ability to inhibit oxidative stress [104] and cholesterol acyltransferase activity [105]. Research results indicate that exposure to enniatins and beauvercin may have a negative impact on reproduction in ruminants. The in vitro study has been shown to inhibit the production of steroid hormones by cattle ovaries [106,107,108]. Contamination of feed for primiparous dairy cows appears to be a factor in udder disease [109].

It has been shown that ENNB and BEA may disrupt immune homeostasis by reducing the ability to recognize pathogens and changing the cytokine profile in the mammary gland microenvironment [17].

Exposure of mammary epithelial cells to ENNB and BEA significantly decreased cell viability in a concentration-dependent manner, with BEA being more toxic than ENNB [109], leading to the loss of milk-secreting cells [17,109]. In the tests carried out on milk samples in Poland, the presence of ENN B and BEA was detected [110] while in Portugal additional concentrations of ENNA, ENNA1 and ENNB1 were detected [111]. Despite the lack of direct health risk for the consumer, the harmful effects of low concentrations of these mycotoxins with long-term exposure cannot be ruled out [110]. However, it should be emphasized that these mycotoxins are not eliminated during milk processing, i.e., sterilization or pasteurization.

Emerging mycotoxins have been shown to have immunomodulatory activity in the innate immune response in ruminants, as evidenced by the effect of ENNB on cattle leukocytes [112,113]. ENNB was shown to inhibit phagocytosis and increase the production of extracellular ROS, leading to a decrease in the herd’s resistance to infections. Additionally, the results of the study indicate a cytotoxic effect of ENNB on intestinal cells in cattle [112]. The effect of emerging mycotoxins on ruminants is still a subject of research; however, no cases of poisoning with these compounds have been reported in cattle.

## 4. The Effects of Mycotoxins on Cattle Immunity

It has been well documented that some mycotoxins can modulate cattle immune response. The in vitro study by Xu et al. [109] showed the immunomodulatory effect of DON, ENNB and BEA within the cattle mammary epithelium. These mycotoxins, however in different ways, modulated the innate immunity of the epithelium by affecting the gene expression of some cytokines, proteins and receptors. In this study, DON effectively stimulated the gene expression of proinflammatory cytokines tumor necrosis factor α (TNF-α) and interleukin 6 (IL-6) as well as transforming growth factor beta (TGF-β), a regulator of inflammatory processes [109,113]. However, this effect was seen after prolonged exposure to the mycotoxin [109]. A significant increase in the milk concentration of TGF-β1 and TGF-β2 was observed following experimental intramammary infection of cows with *Staphylococcus aureus*, the Gram-positive bacteria responsible for mastitis [114]. ENNB showed a distinctly different effect on the cytokine expression, which was characterized by a significant downregulation for IL-6 and TGF-β at different time points of mycotoxin exposure, with no visible effect on TNF-α expression [109]. This study also showed the immunomodulatory effect of the mycotoxins on the gene expression of tight junction (TJ) proteins known as paracellular transport determinants in the mammary epithelium [109,115]. In general, these mycotoxins caused downregulation of gene expression of two of the three proteins tested, such as occludin and claudin 3, which was evident at different time points depending on the mycotoxin type and the time of exposure of the cattle mammary epithelial cell line. The most significant effect was shown for the mRNA expression of claudin 3, in contrast to TJ protein Zonula occludens-1, where none of the mycotoxins caused significant changes in its expression. It may indicate a potential disruption in cell permeability within the cattle mammary epithelium [109]. Both of the tested mycotoxins also had an immunomodulatory effect on changes in the mRNA expression of an important component of an innate immune response, which is Toll-like receptors (TLRs) [109,116]. Toll-like receptor 4 (TLR4) tested in this study can initiate an inflammatory response to some components of Gram-negative mastitis pathogens, including *Escherichia coli* [100,113]. The significant effect was shown only in the case of the longest time point of mycotoxin exposure. At that time point, DON significantly upregulated the expression of TLR4 [109].

Another in vitro study showed the changes in the expression of up-and down-regulated differentially expressed genes in cattle macrophages exposed for shorter and longer periods to sublethal concentrations of OTA and CIT. A general effect of both mycotoxins administered alone and in combination was an impairment of macrophage function in the mycotoxin-treated cells, regardless of the exposure time. In this study, various changes in the macrophage transcriptome were observed de-pending on the type of mycotoxin and the duration of exposure. After shorter exposure, CIT was most able to up-regulation of expression of genes involved in cell death and survival, whereas most altered in the OTA-treated cells were genes associated with gene expression. Most down-regulated genes after exposure to CIT were those involved in cellular function and maintenance. In the OTA-treated cells, a down-regulation of genes associated with cell cycle and cellular assembly was observed, which probably indicates the inhibition of macrophage proliferation. The most visible change in the macrophage transcriptome after prolonged exposure to CIT was an up-regulation of cell death and survival-associated genes, while the most altered genes in OTA-treated cells were those related to DNA replication, recombination and repair. All these processes occurring in the target cells under the effect of OTA are related to DNA damage induction. The mycotoxins showed also an additive or synergistic effect in inhibiting some immune response mechanisms [117].

The study of Gallo et al. [94] demonstrated the effect of different *Fusarium* myco-toxins on the immune system of lactating dairy cows exposed in vivo. A general immunosuppressive effect of the mycotoxins was observed in the significant down regulation of expression of several genes in circulating leukocytes of cows, such as TLR2, MYD88 and IL1R [94]. Toll-like receptor 2 (TLR2) is known as one of pattern recognition receptors which play an important role in the cattle innate immune mechanisms. It has a possibility to recognize and bind some microbial components such as peptidoglycan or lipotechoic acid and mediate the immune response to Gram-positive bacteria [114]. Another important target gene from the point of view of innate immunity was MYD88, which plays an essential role in an immune cell activation through TLRs. A down-regulation of its expression may lead to an impairment of the host defense mechanisms against the pathogen [109,118]. Therefore, a significant decrease in the expression of TLR2, MYD88 and IL1R genes in circulating leukocytes of the exposed lactating dairy cows clearly indicates the immunosuppressive effect of the Fusarium mycotoxins on innate immunity [118].

Another in vivo study demonstrated the immunosuppressive effect of *Fusarium* and *Aspergillus* mycotoxins such as deoxynivalenol, aflatoxin B1 (AFB1) and zearalenone in dairy cows fed with naturally contaminated feed [119]. The long-term exposure of cows to diets with low concentrations of mycotoxins caused a decrease in serum immunoglobulin A (IgA) concentration, a key component of mucosal immunity that serves as the primary defense against pathogens [113,119]. This study was confirmed by a previous in vivo study in which midlactation cows were fed a diet with *Fusarium* mycotoxin contaminants from a natural source. In cows fed the contaminated diet, a significant decrease in serum IgA concentration was also shown. However, in this study, the effect of the contaminated diet on the serum concentration of the other tested Ig classes, i.e., IgM and IgG, was not observed [120]. In another study, an impairment of phagocytic activity of blood neutrophils was observed in mid-lactation cows exposed to a *Fusarium* mycotoxin-contaminated diet [64].

In another study, the changes in some markers of innate immunity were observed in dairy cows fed the AFB1-contaminated diet. Feeding the contaminated diet increased plasma haptoglobin concentration with no significant changes in concentrations of other tested acute phase proteins (APPs) such as ceruloplasmin and fibrinogen [121]. Haptoglobin is one of the indicator APPs in cattle, the increase in which is observed in response to infection or inflammation [122]. Cytometric analysis of neutrophil-adhesin molecules also showed no significant changes after AFB1 dosing; however, some increasing tendency was observed for β2-integrin expression in cows fed the contaminated diet. Other tested markers of innate immunity, i.e., phagocytic neutrophils and neutrophil phagocytic activity, were not affected when the AFB1 toxin diet was fed [121]. For comparison, an in vitro study showed an impairment of phagocytosis, bacterial killing activity and intracellular reactive oxygen species (ROS) generation in the isolated cattle blood neutrophils exposed to very low doses of AFB1. However, the toxin enhanced extracellular ROS generation in the treated neutrophils, which proves the activation of the prooxidant its effect. In contrast, the AFB1 did not affect the viability of cattle neutrophils regardless of the dose used [123].

The study of Jovaišienė et al. [119] showed a positive effect of Anti-Mycotoxin Additive on changes in the immunity status of cows caused by feedborne mycotoxins, expressing by significantly increased serum IgA concentration compared to the control, in which a decrease in this parameter was observed at the end of the study [119]. In another study, supplementation of the contaminated diet with 0.2% polymeric glucomannan mycotoxin adsorbent resulted in a less pronounced reduction in IgA concentration in dairy cows fed with it compared to the diet without this additive [120]. Feeding the AFB1-contaminated diet with the addition of mycotoxin-sequestering agent administered at two doses (low and high) generally prevented the adverse effect of toxin on innate immunity of dairy cows, which manifested in the stabilization of the concentration of some parameters, i.e., haptoglobin [121].

## 5. Mitigation of the Effects of Mycotoxins on Cattle

In clinical practice, chronic mycotoxin poisoning in ruminants is common and is often the result of prolonged feeding of contaminated feed (Table 3). Acute mycotoxin poisoning in cattle is rarely observed.

**Figure 8:**
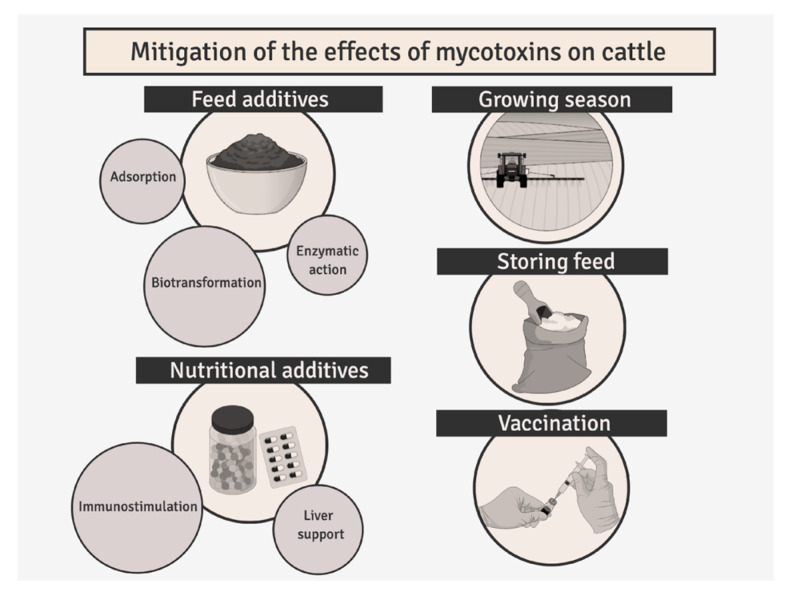
Methods of limiting the effects of mycotoxins on cattle.

To reduce the risk of mycotoxin poisoning, it is essential to control fungal growth both during the growing season and while storing feed [124,125]. This can be achieved through appropriate agronomic practices and various physical, chemical, or biological treatments (Figure 8).

In clinical practice, to prevent mycotoxicoses, animals are often given feed additives that help mitigate the harmful effects of fungal toxins on the body. These additives work through various mechanisms, with the most common being the adsorption of mycotoxins by substances like bentonite, zeolite, or aluminum silicate. These substances are characterized by low chemical reactivity and a large surface area for adsorption. Their absorptive capacity can be further enhanced using nanotechnology.

In addition, some feed additives aimed at combating mycotoxins contain substances that break down or biotransform the toxins. Substances like layered aluminosilicates, chitin biopolymers, and specialized enzymes convert mycotoxins into less toxic compounds, effectively neutralizing them.

An additional factor that enhances the effectiveness of anti-mycotoxin preparations is the inclusion of substances that support liver detoxification or have immunostimulatory properties. The multi-dimensional action of nutritional additives helps significantly reduce the toxic effects of mycotoxins on the animal’s body, leading to a notable improvement in production parameters. Vaccination against aflatoxicosis is a promising method for reducing aflatoxin toxicity, especially concerning its negative impact on farm animals, including dairy cattle. Aflatoxins, particularly their metabolite aflatoxin M1 (AFM1), can pass into milk, posing a risk to human health. Vaccination against aflatoxicosis involves inducing an immune response to aflatoxins in the animal [126,127]. Studies have shown that vaccinating animals with vaccines containing aflatoxin B1 (AFB1) conjugated to appropriate carriers, such as keyhole limpet hemocyanin (KLH), can stimulate the production of antibodies against this mycotoxin. These antibodies can neutralize aflatoxins, reducing their negative impact on animal health and lowering the level of AFM1 in milk. Research on vaccination against aflatoxicosis indicates that such vaccines can reduce the level of AFM1 in milk by up to 46% [126,127]. However, the production of aflatoxicosis vaccines is expensive, making them an impractical option for large-scale livestock farming. Nevertheless, vaccines could be a valuable option in regions where aflatoxin contamination of feed is widespread and poses a serious threat to both animal and human health. By implementing these strategies, the impact of mycotoxins on cattle can be mitigated, reducing the risks to animal health, milk quality, and overall farm productivity.

## 6. Conclusions

Mycotoxins pose a significant threat to cattle health, primarily through disturbances of ruminal fermentation and modulation of the immune system, which may lead to immunosuppression. Their presence in milk and beef also represents a risk to human health. Despite the relatively higher resistance of cattle resulting from rumen microflora activity, animals are frequently exposed to multiple mycotoxins simultaneously, including emerging mycotoxins whose effects are not yet fully understood. In addition, climate change promotes the growth of mycotoxin-producing fungi, increasing the scale of this threat. Therefore, further research and the implementation of effective measures to reduce exposure of both animals and humans to mycotoxins are necessary.

## Figures and Tables

**Table 1:** Maximum levels of total aflatoxins (B1, B2, G1, G2) in feed [25].

Intended Use	Feed	Action Level
Finishing beef cattle	corn and peanut products	300 ppb
Beef cattle	cottonseed meal	300 ppb
Breeding beef cattle	corn and peanut products	100 ppb
Immature animals (sheep and goats less than 4 months of age, cattle less than 6 months of age)	corn, peanut products and other animal food and food ingredients but excluding cottonseed meal	20 ppb

**Table 2:** Maximum levels for certain contaminants in feed [38].

Mycotoxins	Feedstuff	Maximum Level (µg /kg)
Aflatoxin	All feed materials	20
	Complete feedstuffs for beef cattle	20
	Complete feedstuffs for dairy cattle	5
	Complete feedstuffs for calves	10
Deoxynivalenol	Feed materials—Cereals and cereal products	8
	Feed materials—Maize co-products	12
	Complete feedstuffs for beef and dairy cattle	5
	Complete feedstuffs for calves	2
Ochratoxin A	Feed materials—Cereals and cereal products	250
Zearalenone	Feed materials—Cereals and cereal products	2000
	Feed materials—maize co-products	3000
	Complete feedstuffs	500
Fumonisins (sum of B1 and B2)	Feed materials—Cereals and cereal products	60,000
	Complete feedstuffs for dairy and beef cattle	50,000
	Complete feedstuffs for calves	20,000

**Table 3:** The effects of mycotoxins on cattle.

Effects	Mycotoxins	Mechanisms of Action	References
Hepatotoxicity	aflatoxins, ochratoxins, DON, T-2, HT-2, FB1	activation of cytochrome P450, covalent binding of toxin metabolites to DNA, RNA and proteins, formation of tissue adducts that interfere with DNA, RNA and protein synthesis, fatty changes, coagulopathy, inhibition of cell growth and differentiation, inhibition of enzyme activity	[30,36,69,74,91]
Enterotoxicity	trichothecenes (DON, 15-AcDON), T-2, HT-2, ENNB	disturbances in the integration of the intestinal barrier and intestinal structure, enhanced capacity for activate mitogen activated protein kinase, alteration of membrane structure and mitochondrial function, stimulation of lipid peroxidation, hypoxia, oxidative stress, induction of cell death, activation of cytokines and chemokines, morphological and functional damage to the mucous membrane of the intestines, hemorrhagic enteritis, reduction the amount of digestible protein reaching the duodenum, cellular necrosis of intestinal mucosa	[61,62,63,64,74,112]
Nephrotoxicity	ochratoxins, FB1	renal fibrosis, presence of hyaline casts, proximal tubule damage, tubular dilatation, inhibition of cell growth and differentiation, inhibition of enzyme activity	[31,91]
Immunosuppression	aflatoxins, ochratoxins, DON, T-2, HT-2, FB1, FB2, ENNB, BEA	influence on the production of cytokines, non-specific humoral immunity factors such as interferon, complement and certain bectericidal components of blood serum, inhibition of protein, RNA and DNA synthesis, reduction in NK cell activity, induction of oxidative stress, binding to lipids and proteins such as plasma albumin, cellular necrosis, reducing the ability to recognize pathogens and changing the cytokine profile, inhibition of phagocythosis, increased production of extracellular ROS, modulation of innate epithelial immunity by influencing gene expression of some cytokines, proteins and receptors	[17,30,31,47,51,61,62,63,74,93,110]
Carcinogenicity	aflatoxins, ochratoxins, fumonisins		[30,31,36]
Loss of appetite	aflatoxins, DON, T-2, HT-2, fumonisins		[39,74,75,94,95]
Poor weight gain	aflatoxins	altered protein and lipid metabolism in the liver and disturbances in hormonal metabolism	[30,39,40]
Reduced feed convertion	aflatoxins, FB1, FB2		[30,39]
Reduced milk production	aflatoxins, ochratoxins, DON, FB1, FB2, ENNB, BEA	altered protein and lipid metabolism in the liver and disturbances in hormonal metabolism, increased somatic cell count in milk, loss of milk-secreting cells	[17,39,40,56,67,93,94,95,109]
Jaundice	aflatoxins		[39]
Diarrhea	ochratoxins, DON, T-2, HT-2,	impaired intestinal absorption and impaired permeability of nutrients caused by morphological and functional damage to the mucous membrane of the intestines	[55,64,74]
Dehydration	ochratoxins		[56]
Anorexia	aflatoxins, ochratoxins, T-2, HT-2		[40,55]
Coordination disorders	ochratoxins		[55]
Symptoms of uremia	ochratoxins		[58]
Hemorrhages	T-2, HT-2		[74]
Necrosis of mitotically active tissues	T-2, HT-2	e.g., skin, intestinal mucosa, spleen, bone marrow, ovaries, testes	[74]
Increased body temperature	T-2		
Reproductive disorders	aflatoxins, ZEA, ENNB, BEA	necrosis of ovarian and testicular cells, inhibition of steroid hormones production	[36,74,105,107]
Teratogenicity	aflatoxins		[36]
Abortions	T-2, ZEA		[76]
Increased neonatal mortality	T-2		
Fertility disorders	ZEA		
Symptoms of hyperestrogenism	ZEA	interaction with the cytoplasmic receptor that binds to 17b-estradiol and translocation of the receptor site to the nucleus	[82]
Sex hormone disorders	ZEA, ENNB, BEA		
Udder diseases	OTA, ZEA, ENNB, BEA	immunomodulatory effect within mammary epithelium, modulation of innate epithelial immunity by influencing gene expression of some cytokines, proteins and receptors mammary hypertrophy, changing the cytokine profile in the mammary gland microenvironment	[17,108]

## Data Availability

No new data were created or analyzed in this study. Data sharing is not applicable to this article.

## Abbreviations

The following abbreviations are used in this manuscript:

AFB1	aflatoxin B1
ZEA	zearalenone
EU	European Union
IgA	immunoglobulin A
DON	deoxynivalenol
OTA	ochratoxin A
FB	fumonisin
IARC	International Agency for Research on Cancer
CYP	cytochrome P450
DNA	deoxyribonucleic acid
RNA	ribonucleic acid
FDA	Food and Drug Administration
DDGs	dried distillers grains
NK	natural killer cells
DAS	diacetoxyscirpenol
MAS	monoacetoxyscirpenol
NEO	neosolaniol
NIV	nivalenol
3-Ac-DON	3-acetyldeoxynivalenol
15-Ac-DON	15-acetyldeoxynivalenol
ENN	enniatins
BEA	beauvericin
FP	fusaproliferin
MON	moniliformin
AUR	aurofusarin
MPA	mycophenolic acid
EMO	emodin
TNF-α	tumor necrosis factor α
IL-6	interleukin 6
TGF-β	transforming growth factor beta
TJ	tight junction proteins
TLRs	Toll-like receptors
ROS	reactive oxygen species
KLH	keyhole limpet hemocyanin
